# Time/Movement Estimation and Mental Rotation Tasks as Early Cognitive Markers in Alzheimer's Disease

**DOI:** 10.1002/brb3.71077

**Published:** 2025-11-19

**Authors:** Vaiva Sutnikiene, Gyte Pakulaite‐Kazliene, Egle Audronyte, Justina Kuzmickaite, Gintaras Kaubrys

**Affiliations:** ^1^ Faculty of Medicine Institute of Clinical Medicine Clinic of Neurology and Neurosurgery Vilnius University Vilnius Lithuania

**Keywords:** Alzheimer's disease, mental rotation task, mild cognitive impairment, Time–Wall task

## Abstract

**Introduction:**

Cognitive impairments, including memory decline and executive dysfunction, are well‐documented in Alzheimer's disease (AD); however, distortions in temporal judgment, motion perception and mental rotation in the early stages remain underexplored. Existing research has predominantly relied on verbal time‐estimation tasks, with a limited investigation into alternative paradigms, such as time reproduction or bisection tasks. This study investigated the diagnostic utility of time–movement estimation and mental rotation tasks from the psychology experiment building language (PEBL) test battery for identifying early cognitive impairment. Moreover, it assessed correlations among task performance, cognitive test scores, and demographic variables.

**Methods:**

This cross‐sectional study included 28 patients with mild dementia (MD), 27 with amnestic mild cognitive impairment (MCI), and 26 with normal cognitive function as the control cohort (CC). Participants completed the mini‐mental state examination, clinical dementia rating assessments, Alzheimer's disease assessment scale–cognitive subscale 13 (ADAS–Cog 13), and PEBL‐based Time–Wall and mental rotation tasks.

**Results:**

Time–Wall task inaccuracy scores exhibited strong diagnostic accuracy in distinguishing between the CC and early AD (MCI and MD), with an AUC of 0.9, and effectively differentiated CC from MCI, with an AUC of 0.86. Conversely, the mental rotation task exhibited weaker diagnostic properties, with AUC values of 0.75 for distinguishing CC from early AD and 0.71 for distinguishing CC from MCI. The multinomial logistic regression model accurately categorized 75.3% of participants (CC = 92.3%, MCI = 59.3%, and MD = 75%), utilizing demographic data and ADAS–Cog 13 and Time–Wall inaccuracy scores as predictors. Both ADAS–Cog 13 and Time–Wall task inaccuracy scores were statistically significant predictors (*X* ^2^ = 49.41, *p* < 0.001; *X* ^2^ = 9.24, *p* = 0.01, respectively). Time–Wall task inaccuracy scores did not notably correlate with age.

**Conclusions:**

The Time–Wall task showed strong diagnostic utility in identifying early AD, independent of age. The mental rotation task exhibited low sensitivity and requires further investigation regarding its potential to reflect compensatory brain network functions.

## Graphical abstract

This study evaluated time–movement estimation and mental rotation tasks as diagnostic tests for early Alzheimer's disease using freely available tests from the PEBL battery. Moreover, correlations between cognitive test outcomes and demographic variables were examined.

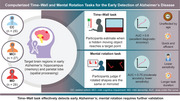

## 1 Background

Alzheimer's disease (AD) is the most common cause of dementia, globally affecting over 57 million individuals (WHO 2025)]. Its prevalence increases markedly with age: approximately 13.2% and 33.4% of individuals aged 75–84 years and those aged ≥85 years are affected by AD, respectively (Rajan et al. 2021). The early stages of the disease, frequently accompanied by subtle clinical symptoms, constitute a critical window for intervention and care planning, during which treatment may be most beneficial (Spargo et al. 2023). Contemporary biological research frameworks for AD incorporate in vivo biomarkers of β‐amyloid and tau, thereby extending the neuropathological definition of the disease to include living individuals (Jack et al. 2024). The identification of subtle cognitive impairment or preserved cognitive function in older adults is highly dependent on the sensitivity and specificity of the assessment tools employed (Kessels 2019).

In the domain of neuropsychological assessment, computerized evaluations offer a rapid and efficient means of appraising cognitive function [6]. Computers facilitate standardized instruction delivery across multiple languages and support the collection of large volumes of objective data while minimizing experimenter‐related errors (Kessels 2019; Chan et al. 2018). Compared with traditional paper‐and‐pencil methods, computerized assessments can be seamlessly integrated into neuroimaging settings and considerably reduce the manual effort attributed to scoring, analysis, and longitudinal monitoring of cognitive performance (Chan et al. 2018; Kochan et al. 2022).

An increasing number of computerized cognitive assessments are currently commercially available and can be self‐administered at home (Kochan et al. 2022). Nonetheless, the cost of extensively researched validated test batteries may limit their accessibility in primary care settings and among researchers in developing countries (Piper et al. 2015). By contrast, the psychology experiment building language (PEBL) is freely available for download at http://pebl.sourceforge.net/ and is accompanied by comprehensive documentation, enabling researchers to customize tests for their specific experimental requirements. The current 2.1 version of the PEBL battery includes approximately 50 assessments, encompassing many widely recognized neuropsychological measures (Piper et al. 2015; The PEBL Project 2019).

The Time–Wall task is a nonverbal assessment designed to evaluate temporal judgment, motion perception, and decision‐making abilities (Piper et al. 2015; The PEBL Project 2019). Its original purpose was to measure an individual's ability to predict the time required for a target to move vertically at a constant speed over a specified distance. This task engages cognitive processes involved in both motion perception and temporal judgment (Piper et al. 2015; The PEBL Project 2019; Kim et al. 2024). The association between temporal judgment and memory decline in AD is believed to involve the hippocampus, a brain region essential for both functions and particularly vulnerable to AD‐related pathology (El Haj and Kapogiannis 2016).

Numerous studies have indicated that patients with AD experience difficulties in motion processing and visuospatial disorientation, implicating dysfunction in the posterior parietal regions of the dorsal visual pathway (Liu et al. 2019; Deng et al. 2016). The dorsal stream hypothesis of AD is further supported by consistent findings of parietal atrophy and reduced activity in the parietal lobe of affected individuals (Liu et al. 2019; Deng et al. 2016). Consequently, tasks assessing temporal judgment and motion perception may serve as valuable diagnostic tools for AD's early detection.

The mental rotation task is employed to evaluate visuospatial abilities. Recent studies have shown its applicability in patients with mild cognitive impairment (MCI) and AD, particularly in evaluating non‐amnestic symptoms (Piper et al. 2015; Suzuki et al. 2018). The task involves mentally transforming the visuospatial features of an image until it matches a target without any physical movement. Performance is measured based on response accuracy and time, which increases with the rotation angle, reflecting greater difficulty as the angles become larger (Piper et al. 2015; Suzuki et al. 2018; Langner et al. 2023). Lineweaver et al. found that patients with early AD tend to perform poorly on mental rotation tasks, potentially due to pathological changes in the parietal and temporal lobe association cortices, which impair spatial processing (Lineweaver et al. 2005).

Despite extensive research on cognitive impairments in AD, particularly regarding memory decline and executive dysfunction, distortions in temporal judgment and motion perception in early AD have received comparatively little attention (El Haj and Kapogiannis 2016). Temporal processing and memory impairments are commonly associated with hippocampal dysfunction. Emerging evidence highlights the hippocampus as essential for differentiating time intervals, organizing temporal sequences, and encoding the flow of time (MacDonald et al. 2014; Meck et al. 2013). However, most studies to date have focused on verbal time‐estimation tasks, with a limited investigation into alternative paradigms, such as time reproduction or bisection tasks (El Haj and Kapogiannis 2016).

Consequently, this study evaluated time–movement estimation and mental rotation tasks as diagnostic tests for early AD using freely available tests from the PEBL battery. Moreover, correlations between cognitive test outcomes and demographic variables were examined.

## 2 Methods

### 2.1 Ethical Considerations and Informed Consent

This study was approved by the Vilnius Regional Bioethics Committee on January 11, 2022 (approval number: 2022/1‐1405‐877). Moreover, this study was conducted in accordance with the principles of the Declaration of Helsinki and standards for reporting diagnostic accuracy studies (STARD) guidelines. Written informed consent was obtained from all participants prior to study participation and for the publication of their de‐identified data.

### 2.2 Study Design

In this cross‐sectional study, participants were recruited from the memory clinic at Vilnius University Hospital, Santaros Klinikos. A cognitive neurologist diagnosed probable AD based on established criteria (Albert et al. 2011), further supported by observed cognitive decline, indicating an ongoing pathological process. Of the 28 patients with mild dementia (MD), seven (25%) had medical records with documented positive AD cerebrospinal fluid (CSF) biomarkers, consistent with the 2018 National Institute on Aging and Alzheimer's Association (NIA‐AA) research framework for AD diagnosis (Jack et al. 2018). Given the exploratory nature of our study, the analysis of CSF biomarkers was not conducted within the scope of this research.

The diagnosis of amnestic MCI attributed to AD was established using clinical and cognitive criteria, excluding vascular, traumatic, and other medical etiologies of cognitive decline, and was supported by documented evidence of progressive cognitive deterioration (McKhann et al. 2011). Structural magnetic resonance imaging confirmed neuronal damage in all MCI cases, with positive AD CSF biomarkers documented in eight of 27 participants (30%), confirming an AD diagnosis based on the 2018 research framework (Jack et al. 2018).

Exclusion criteria were as follows: (1) neurological disorders affecting the central nervous system, apart from MCI and MD; (2) cerebrovascular conditions with Hachinski ischemic scores ≥ 4; (3) a history of head trauma; (4) major psychiatric disorders, including schizophrenia, delirium, psychosis, or depression (with a geriatric depression scale score > 9); (5) sensory impairments in vision or hearing that could interfere with cognitive assessments; (6) major cardiovascular, hepatic, or metabolic diseases; (7) substance abuse; and (8) the use of psychoactive medications.

The study included three cohorts: 28 participants with MD, 27 with amnestic MCI, and 26 older adults with normal cognitive abilities who served as the control cohort (CC). Participants in the CC reported no cognitive impairments, achieved a clinical dementia rating total score (CDR‐TS) of 0, and exhibited no neurological abnormalities. Participants with MCI met the clinical and cognitive criteria for MCI due to AD, as defined by the NIA/AA [17], and were assigned a CDR‐TS of 0.5. Participants in the MD cohort fulfilled the NIA/AA criteria for probable AD [18] and had a CDR‐TS score of 1.

### 2.3 Assessments of Cognitive Function

The mini‐mental state examination (MMSE) was employed to assess general cognitive function. The clinical dementia rating sum of boxes (CDR‐SB) scale was used to evaluate the severity of functional impairment and cognitive decline. Moreover, participants completed the AD assessment scale–cognitive subscale (ADAS–Cog) 13 for a comprehensive cognitive evaluation. This test includes components such as word recall, following commands, constructional praxis, delayed recall, naming, ideational praxis, orientation, word recognition, remembering test instructions, understanding spoken language, word‐finding difficulty, spoken‐language ability, and number cancellation. ADAS–Cog 13 scores ranged from 0 to 85, with higher scores indicating greater cognitive impairment.

### 2.4 Assessment of PEBL Battery Tests: Time–Wall and Mental Rotation Tasks

**FIGURE 1:**
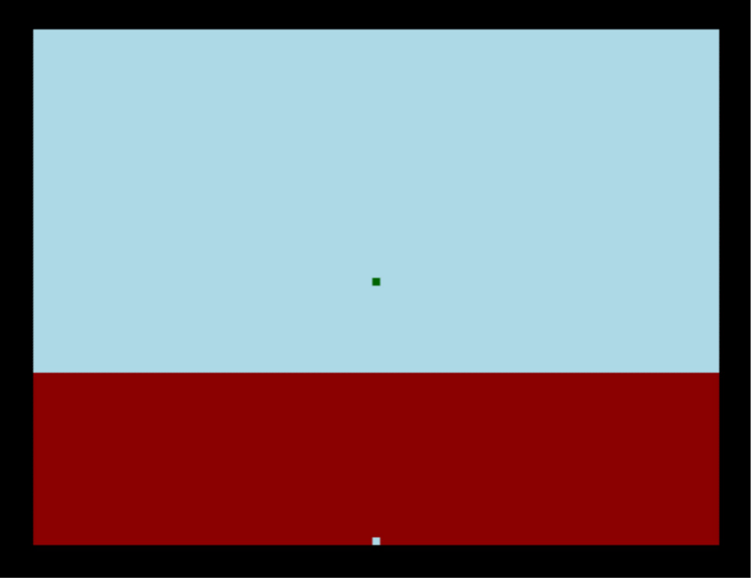
Illustration of the Time–Wall task. Participants estimate when a moving object (green square), traveling at a constant speed, will reach a gap (white square at the bottom) after becoming obscured by a red wall.

The Time–Wall task is a time–movement estimation exercise in which a moving object disappears behind a wall, and participants must judge when it will reach a visible gap, assuming constant speed throughout the trial. After descending two‐thirds of its trajectory, the target moves behind a wall and consequently becomes obscured from view (Figure 1). This task was adapted from the Unified Tri‐Services Cognitive Performance Assessment Battery (Systems Research Laboratories Inc., Dayton, Ohio, USA) (Sam et al. 2013). Participants were instructed to press the space bar at the moment they believed the target aligned with a notch at the bottom of the screen. The task comprised 20 trials, each lasting between 2 and 10 s.

The average error time across trials was used to evaluate each participant's precision in time perception. Response inaccuracy was calculated as the percentage difference between the response time (RT) and target time (TT) using the formula:

(1) $$\left(\mathrm{RT}-\mathrm{TT}\right)/\mathrm{TT}\times 100.$$

Additionally, the percentage of late responses was computed to further characterize temporal estimation performance.

**FIGURE 2:**
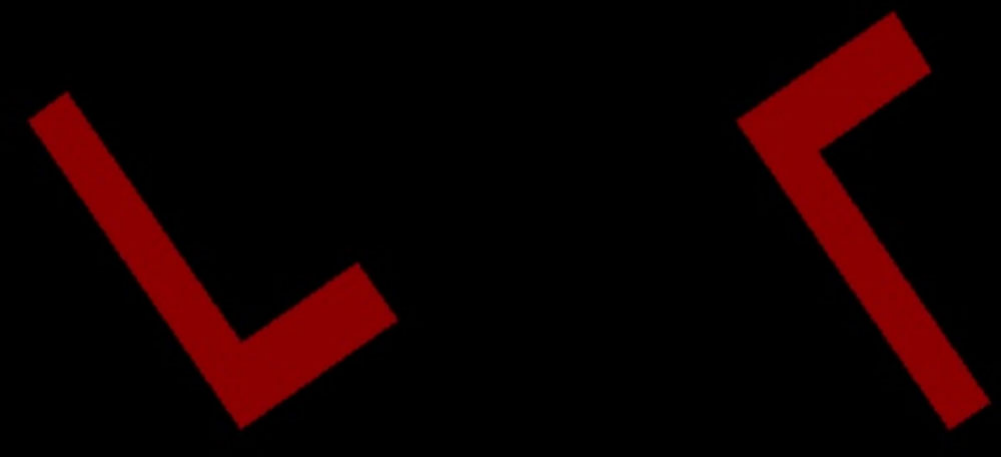
Example of mirror images is depicted. Participants are required to determine whether two figures on the screen's plane are identical or mirror images, with the figures rotated relative to each other.

The mental rotation task is a two‐dimensional adaptation of the classical test introduced by Shepard (Langner et al. 2023; Shepard and Metzler 1971). Participants were instructed to press either the “D” or “S” key on the keyboard to indicate whether a pair of red shapes displayed on a gray screen was mirrored on the screen's plane (mirror condition). The shapes were rotated relative to each other at angles of 0°, 45°, 90°, 135°, and 180° (angle conditions). The pairs consisted of a familiar shape (an L shape) and an unfamiliar shape (a bolt) (Figure 2). The task was modified and included six practice trials and 32 experimental trials, each with a maximum duration of 45 s, resulting in a total task duration of up to 30 min. Correct responses and reaction times were recorded for each trial.

### 2.5 Data Analysis

To ensure adequate power, we calculated the required sample size using G*Power for Windows version 3.1 (Heinrich Heine University Düsseldorf, Düsseldorf, Germany), setting power at 0.85 and significance at *p* < 0.05 (one‐tailed), while anticipating a large effect size (*f* = 0.4). Consequently, the Kruskal–Wallis test indicated that a minimum of 72 participants was required.

The Shapiro–Wilk test was used to assess the normality of data distribution. Differences in demographic, clinical, and test outcome data among cohorts were evaluated using the Kruskal–Wallis or chi‐square tests. Spearman's rank correlation coefficient was applied to examine correlations between variables. Linear regression models, adjusted for demographic factors, were subsequently used to predict continuous dependent variables. For each model, we reported the coefficient of determination (*R* ^2^) to indicate the proportion of variance explained by the model, the F‐statistic (F) to assess overall model significance, and the standardized beta coefficient (β) to reflect the strength and direction of the correlations between predictors and outcomes. Multinomial logistic regression was conducted to examine the correlation between predictor variables and cohort membership across three categories: CC, MCI, and MD. Nagelkerke *R* ^2^ was used to assess the explanatory power of logistic regression models. Diagnostic performance was assessed using the receiver operating characteristic (ROC) curve analysis. Statistical significance was set at *p* < 0.05, with Bonferroni correction applied to *post hoc* comparisons.

## 3 Results

### 3.1 Participant Characteristics

Analysis revealed no statistically significant differences in sex distribution among the three cohorts (*p* > 0.05). Similarly, no statistically significant differences were found in age, educational background, depressive symptoms, or Hachinski ischemic scores (*p* > 0.05). However, statistically significant differences were observed in cognitive test scores across all three cohorts (*p* < 0.05). Table 1 presents a detailed summary of the demographic and clinical characteristics, as well as the cognitive test performances, of the study participants.

**TABLE 1:** Overview of participant characteristics.

	CC (*n* = 26)	MCI (*n* = 27)	MD (*n* = 28)	Statistic (*χ* ^2^ [2]/H(2), *p*)
Female (%)*	12 (46%)	19 (70%)	18 (64%)	3.09, 0.21
Age*	73 (9)	75 (11)	77 (9)	4.63, 0.10
Years of education*	16 (1)	15 (4)	15 (3)	4.37, 0.11
GD*	4 (2)	5 (1)	4 (2)	1.40, 0.50
HIS*	1 (1)	1 (1)	1 (1)	1.84, 0.40
MMSE**	29 (1)	25 (3)	22 (2)	72.02, < 0.001
ADAS–Cog 13**	12.5 (5.67)	22 (7.66)	31 (7.5)	74.26, < 0.001
CDR‐SB**	0 (0)	1.5 (1)	4.5 (0.5)	53.07, < 0.001

Unless otherwise specified, the data are shown as medians and interquartile ranges.

Statistical significance is set at *p* < 0.05.

Abbreviations: ADAS–Cog 13 = Alzheimer's disease assessment scale–cognitive subscale 13; CC = control cohort; CDR‐SB = clinical dementia rating sum of boxes; GDS = geriatric depression scale; HIS = Hachinski ischemic score; MCI = mild cognitive impairment;MD = mild dementia; MMSE = mini‐mental state examination.

*Cohorts do not differ statistically significantly.

**Three cohorts differ statistically significantly.

### 3.2 Time–Wall Task Results

Statistically significant differences were observed in the Time–Wall task inaccuracy scores [medians and interquartile ranges: CC = 11.46 (9), MCI = 19.43 (10.49), MD = 22.82 (14.35); H(2) = 34.74; *p* < 0.001], with the CC exhibiting lower scores than did both the MCI and MD cohorts. No statistically significant differences were found between the MCI and MD cohorts (*p* = 0.106).

The percentages of late responses are presented as medians and interquartile ranges: CC = 10 (18.75), MCI = 20 (22), and MD = 25 (23) [H(2) = 7.3; *p* = 0.026]. Statistically significant differences were observed between the CC and MD cohorts (*p* = 0.01); however, no statistically significant differences were found between the CC and MCI cohorts (*p* = 0.153) or between the MD and MCI cohorts (*p* = 0.143).

In the study population, Time–Wall task inaccuracy scores were strongly correlated with the MMSE results (Spearman's rho = −0.628; *p* < 0.001) and moderately correlated with CDR‐SB (Spearman's rho = 0.568; *p* < 0.001) and ADAS–Cog 13 (Spearman's rho = 0.594; *p* < 0.001) scores. A weak correlation with education (Spearman's rho = −0.314; *p* = 0.004) (Figure S1) and no notable correlation with age were found (Spearman's rho = 0.17; *p* = 0.129).

Multiple linear regression models, including age, sex, education, and cognitive test scores, as independent variables, were used to determine their predictive values for the Time–Wall task inaccuracy scores. Statistical significance was observed in the following models: MMSE [*R* ^2^ = 0.345, *F* = 11.52, *β* = −0.577, *p* < 0.001]; ADAS–Cog 13 (*R* ^2^ = 0.321, *F* = 10.46, *β* = 0.577, *p* < 0.001), and CDR‐SB (*R* ^2^ = 0.262, *F* = 8.09, *β* = 0.498, *p* < 0.001).

### 3.3 Diagnostic Characteristics of the Time–Wall Task

**FIGURE 3:**
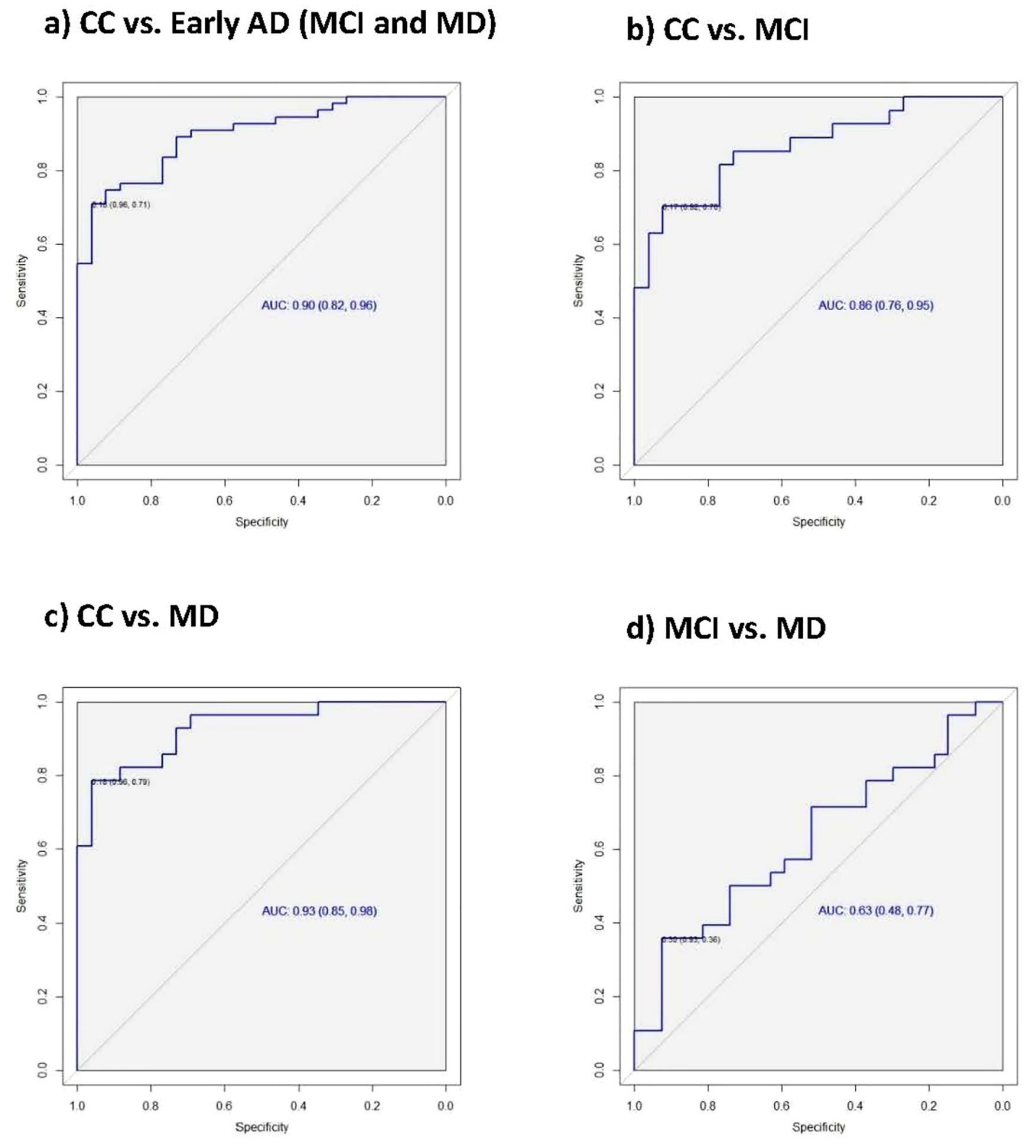
Time–Wall task performance: differentiating between participants with Alzheimer's disease and those in the CC. Abbreviations: CC = control cohort; MD = mild dementia; MCI = mild cognitive impairment.

The efficacy of the Time–Wall task inaccuracy scores in distinguishing participants with early AD from those in the CC was assessed using the ROC curve analysis. The ROC curve analysis revealed strong diagnostic capabilities for differentiating between the CC and early AD (MCI and MD), with an area under the curve (AUC) of 0.9. Similarly, differentiation between the CC and MCI cohorts resulted in an AUC of 0.86. Figure 3 illustrates the ROC curves used to evaluate the Time–Wall task performance.

A threshold of ≥ 18.35 for the Time–Wall task inaccuracies was selected to indicate AD. By applying this threshold to distinguish participants with AD (MCI and MD) from participants in the CC, the Time–Wall task demonstrated a sensitivity of 70.91% (95% CI: 57.1–82.37) and specificity of 96.15% (95% CI: 80.36%–99.9%). Table 2 provides a comprehensive overview of the diagnostic characteristics of the Time–Wall task for differentiating between participants with AD and those in the CC.

**TABLE 2:** Time–Wall task performance: Distinguishing participants with Alzheimer's disease (AD) and control cohort (CC) members.

	CC vs. early AD (MCI and MD): cut‐off ≥ 18.35	CC vs. MCI: cut‐off ≥ 15.08	CC vs. MD: cut‐off ≥ 16.28	MCI vs. MD: cut‐off ≥ 21.67
Sensitivity	70.91% (57.1–82.37%)	77.78% (57.74–91.38%)	82.14% (63.11–93.94%)	57.14% (37.18–75.54%)
Specificity	96.15% (80.36–99.9%)	76.92% (56.35–91.03%)	88.46% (69.85–97.55%)	59.26% (38.80%–77.61%)
Negative predictive value	60.98% (50.66–70.39%)	76.92% (61.48–87.44%)	82.14% (67.25–91.15%)	57.14% (43.97–69.37%)
Positive predictive value	97.5% (84.99–99.63%)	77.78% (62.78–87.90%)	88.46% (72.28–95.75%)	59.26% (45.46–71.73%)
Overall diagnostic accuracy	79.01% (68.54–87.27%)	77.36% (63.79–87.72%	85.19% (72.88–93.38%).	58.18% (44.11–71.35%)

Data are presented as percentages and 95% confidence intervals.

Abbreviations: MCI = mild cognitive impairment; MD = mild dementia.

Multinomial logistic regression was conducted to examine the correlation between predictor variables and cohort membership across three categories: CC, MCI, and MD. A model was first developed that incorporated age, education, sex, and ADAS–Cog 13 scores as predictor variables. This model showed a better fit when compared with a model with only the intercept (*X* ^2^ = 87.85, *p* < 0.001; Nagelkerke *R* ^2^ = 0.745). The model achieved an overall classification accuracy of 76.5%, with correct classification rates of 92.3%, 66.7%, and 71.4% for CC, MCI, and MD, respectively. The ADAS–Cog 13 score was identified as the most robust and statistically significant predictor (*X* ^2^ = 78.20, *p* < 0.001).

The Time–Wall task scores were included in the initial model. The model comprising age, education, sex, ADAS–Cog 13 scores, and Time–Wall task inaccuracy scores showed a marked improvement in fit over the null model (*X* ^2^ = 96.69, *p* < 0.001; Nagelkerke *R* ^2^ = 0.784). The model achieved an overall classification accuracy of 75.3%, with correct classification rates of 92.3%, 59.3%, and 75% for CC, MCI, and MD, respectively. ADAS–Cog 13 and Time–Wall task inaccuracy scores were statistically significant predictors (*X* ^2^ = 49.41, *p* < 0.001; *X* ^2^ = 9.24, *p* = 0.01, respectively).

### 3.4 Mental Rotation Task

Mental rotation task accuracy scores differed statistically significantly among the cohorts [medians and interquartile ranges: CC = 26.5 (4), MCI = 22 (9), and MD = 21.5 (9); *p* = 0.001]. *Post hoc* comparisons revealed statistically significant differences between the CC and both MCI (*p* = 0.007) and MD cohorts (*p* < 0.001), yet not between the MCI and MD cohorts (*p* = 0.094). Moreover, response times varied statistically significantly among the cohorts [H(2) = 15.12, *p* = 0.001; medians and interquartile ranges: CC = 6309.7 ms (4531.4); MCI = 12 684.5 ms (7974.7); and MD = 11 657.3 ms (7053)].

Analysis of the CC, MCI, and MD cohorts revealed no statistically significant differences in accuracy scores or between the response time and sex. Regarding the CC, accuracy scores were similar between males [27.5 (5)] and females [25.5 (3)] (*p* = 0.494), and response times did not differ statistically significantly [males = 6017.9 (6908.05); females = 7235.88 (2957.87); *p* = 0.374]. Regarding the MCI cohort, accuracy scores were comparable between males [23 (13)] and females [22 (7)] (*p* = 0.897), while response times showed no statistically significant difference [males = 6975.6 (8787.42); females = 13080.63 (5720.03); *p* = 0.051]. Accuracy scores for the MD cohort revealed showed no statistically significant sex differences [males = 22.5 (7); females = 18.5 (10); *p* = 0.072]. Similarly, response times did not differ statistically significantly between males [9507.59 (8206.03)] and females [12508.13 (9802.41)] (*p* = 0.08).

In this study population, accuracy scores regarding the mental rotation task revealed a moderate correlation with MMSE (Spearman's rho = 0.472; *p* < 0.001) and ADAS–Cog 13 scores (Spearman's rho = −0.407; *p* < 0.001), and a weak correlation with CDR‐SB (Spearman's rho = −0.372; *p* = 0.001), age (Spearman's rho = −0.247; *p* = 0.026), and education (Spearman's rho = 0.238; *p* = 0.033). Moreover, mental rotation task response times were moderately correlated with MMSE scores (Spearman's rho = −0.405; *p* < 0.001). Weaker correlations were observed with ADAS–Cog 13 (Spearman's rho = 0.386; *p* = 0.001) and CDR‐SB scores (Spearman's rho = 0.277; *p* = 0.015). No notable correlation was found between age (Spearman's rho = 0.19; *p* = 0.09) and the educational level (Spearman's rho = 0.20; *p* = 0.06) (Figure S2).

Multiple linear regression models were used to assess the predictive value of age, sex, education, and cognitive test scores as independent variables for mental rotation task accuracy scores. Statistically significant results were observed for the following models: MMSE (*R* ^2^ = 0.265, *F* = 8.22, *β* = 0.438, *p* < 0.001), ADAS–Cog 13 (*R* ^2^ = 0.178, *F* = 5.32, *β* = 0.334, *p* = 0.001), and CDR‐SB (*R* ^2^ = 0.177, *F* = 5.3, *β* = −0.32, *p* = 0.001).

### 3.5 Diagnostic Characteristics of the Mental Rotation Task

**FIGURE 4:**
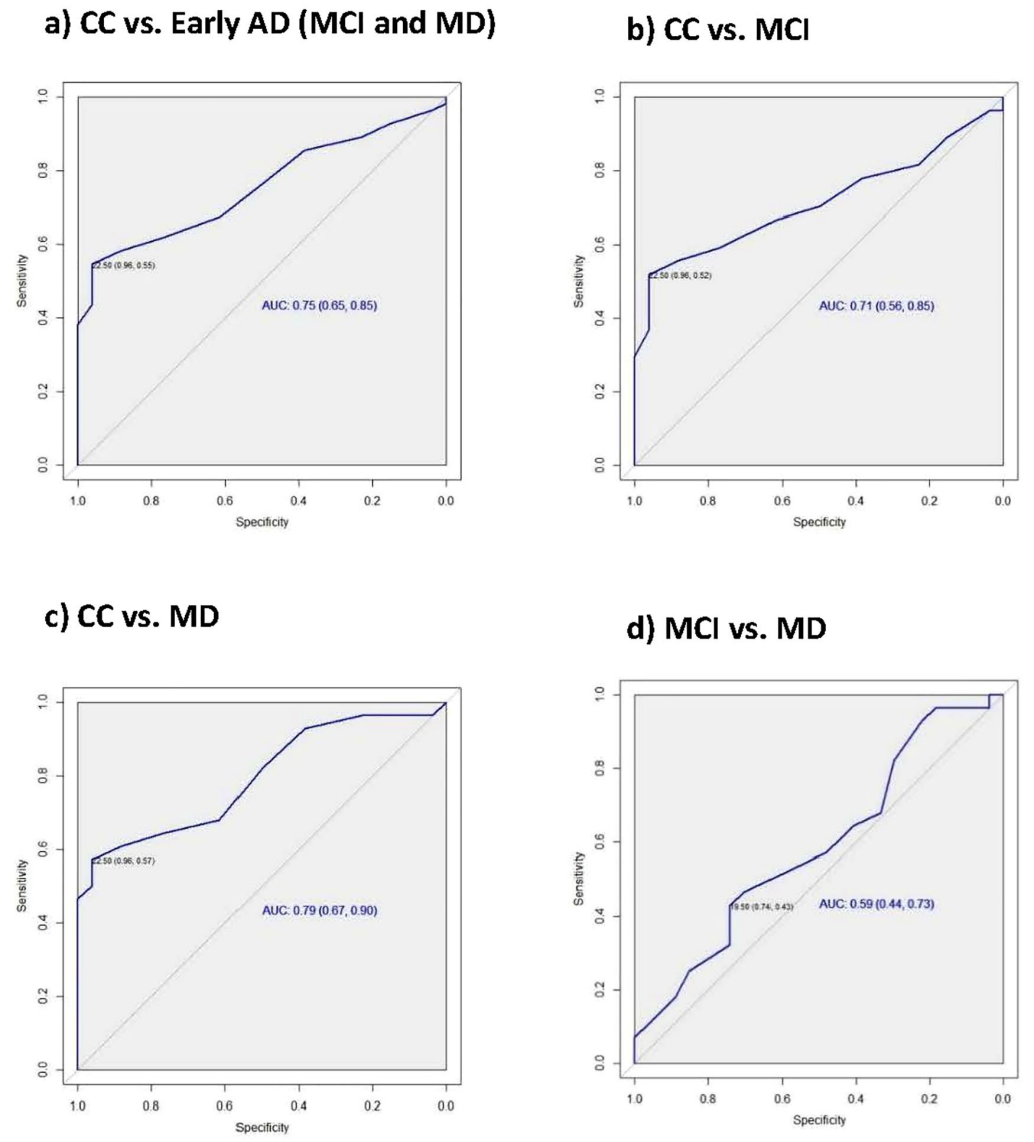
Mental rotation task performance: differentiating between participants with Alzheimer's disease (AD) and cognitively healthy controls. Abbreviations: MD = mild dementia; MCI = mild cognitive impairment; CC = control cohort.

The ability of mental rotation task accuracy scores to differentiate participants with early AD from cognitively healthy controls was evaluated using the ROC curve analysis. The task demonstrated moderate diagnostic accuracy in distinguishing participants in the CC from those with AD (MCI and MD), with an AUC of 0.75. Similarly, differentiation between the CC and MCI cohorts yielded an AUC of 0.71. ROC curves illustrating task performance are presented in Figure 4.

A threshold score of ≤ 22 was selected to indicate AD. Using this cutoff to distinguish participants with AD (MCI and MD) from cognitively healthy controls, the mental rotation task yielded a sensitivity of 54.55% (95% CI: 40.55–68.03) and specificity of 96.15% (95% CI: 80.36%–99.9%). Table 3 presents a comprehensive overview of the diagnostic performance of the mental rotation task in differentiating participants with AD from those in the CC.

**TABLE 3:** Mental rotation task performance: Differentiating between participants with Alzheimer's disease (AD) and cognitively healthy controls.

	CC vs. early AD (MCI and MD): cut‐off ≤ 22	CC vs. MCI: cut‐off ≤ 22	CC vs. MD: cut‐off ≤ 22	MCI vs. MD: cut‐off ≤ 19
Sensitivity	54.55% (40.55–68.03)	51.85% (31.95–71.33%)	57.14% (37.18–75.54%)	42.86% (24.4–662.82%)
Specificity	96.15% (80.36–99.9%)	96.15% (80.36–99.9%)	96.15% (80.36–99.9%)	74.07% (53.72–88.89%)
Negative predictive value	50% (42.57–57.43%)	65.79% (56.34–74.13%)	67.57% (57.43–76.29%)	55.56% (45.82–64.88%)
Positive predictive value	96.77% (81.22–99.52%)	93.33% (66.44%–99%)	94.12% (69.51–99.12%)	63.16% (44.31–78.7%)
Overall diagnostic accuracy	67.9% (56.6–77.85%)	73.58% (59.67–84.74%)	75.93% (62.36–86.51%)	58.18% (44.11–71.35%)

Data are presented as percentages and 95% confidence intervals.

Abbreviations: CC = control cohort; MCI = mild cognitive impairment; MD = mild dementia.

The accuracy score and response time for the mental rotation task were included in the initial model. The model, which incorporated age, education, sex, ADAS–Cog 13 scores, mental rotation task accuracy scores, and response time, showed a marked improvement in fit over the null model (*X* ^2^ = 93.72, *p* < 0.001; Nagelkerke *R* ^2^ = 0.771). The model achieved an overall classification accuracy of 75.3%, correctly classifying 88.5%, 63%, and 75% of the CC and MCI and MD cohorts, respectively. ADAS–Cog 13 was the only statistically significant predictor (*X* ^2^ = 64.01, *p* < 0.001).

## 4 Discussion

In this study, computerized time–movement estimation (Time–Wall) and mental rotation tasks were evaluated as potential diagnostic tools for early AD. The Time–Wall task demonstrated strong diagnostic performance, with AUC values of 0.9 for distinguishing cognitively healthy controls from participants with early AD (MCI and MD) and 0.86 for differentiating the CC from the MCI cohort alone. Conversely, the performance of the mental rotation task was more modest, with AUC values of 0.75 for distinguishing participants in the CC from those with early AD and 0.71 for differentiating the CC from the MCI cohort. Table 4 presents a comparison of Time–Wall and mental rotation administration time and performance with existing cognitive screening tools, differentiating between participants with AD and cognitively healthy controls.

**TABLE 4:** Comparison of Time–Wall and mental rotation administration time and performance with existing cognitive screening tools, differentiating between participants with Alzheimer's disease (AD) and cognitively healthy controls.

Cognitive assessment tool	Administration time	CC vs. MD	CC vs. MCI
MMSE	10–15 min	Sensitivity 81% (78%–84%) Specificity 89% (87%–91%) (Tsoi et al. 2015)	Sensitivity 89.2% (85.4–92.4%) Specificity 45.1% (39.2–51.1%) (Mitchell et al. 2009)
MoCA*	10–15 min	Sensitivity 91% (84%–95%) Specificity 81% (71%–88%) (Tsoi et al. 2015)	Sensitivity 89% (84%–92%) Specificity 75% (62%–85%) (Tsoi et al. 2015)
ADAS‐Cog	30–45 min	Sensitivity 92.2% (88.2–95.5%) Specificity 90.7% (88.8–92.5%) (Wang et al. 2023)	Sensitivity 86.9% (80.0–92.9%) Specificity 83.5% (80.3–86.6%) (Wang et al. 2023)
Time–Wall task	3–5 min	Sensitivity 82.14% (63.11–93.94%) Specificity 88.46% (69.85–97.55%)	Sensitivity 77.78% (57.74–91.38%) Specificity 76.92% (56.35–91.03%)
Mental rotation task**	5–20 min (time limit 30 min)	Sensitivity 57.14% (37.18–75.54%) Specificity 96.15% (80.36–99.9%)	Sensitivity 51.85% (31.95–71.33%) Specificity 96.15% (80.36–99.9%)

Data are presented as percentages and 95% confidence intervals.

Abbreviations: ADAS–Cog = Alzheimer's disease assessment scale–cognitive subscale; CC = control cohort; MCI = mild cognitive impairment; MD = mild dementia; MMSE = mini‐mental state examination; MoCA = Montreal cognitive assessment.

*Not performed in this study.

**The PEBL mental rotation task was modified and included six practice trials and 32 experimental trials.

This study's findings indicated that the Time–Wall task performance was already impaired at the MCI stage, with no marked differences observed between the MCI and MD cohorts. In our study population, the median inaccuracy in the CC was 11.46%. In comparison, Piper et al. have reported an average inaccuracy of 12.3% ± 0.8% across all age cohorts, with a general tendency to respond too early (Piper et al. 2015). Notably, their study found no evidence of performance decline among older adults [8]. These results suggest that the general reduction in processing speed typically associated with aging may confer a slight advantage in this simple decision‐making task, which contrasts with findings from other executive function tests (Piper et al. 2015).

Our study found that inaccuracy scores of the Time–Wall task were strongly correlated with cognitive assessment outcomes, showed a weak correlation with educational background, and no perceptible correlation with age. Furthermore, the Time–Wall task accuracy score emerged as a major predictor in the multinomial logistic regression model used to classify participants into the CC and MCI and MD cohorts. Therefore, these findings highlight the robust diagnostic potential of the Time–Wall task for early AD, with minimal influence from demographic factors, such as age.

While numerous studies have examined cognitive impairments in AD, particularly memory loss and executive dysfunction, relatively few have investigated distortions in temporal judgment and motion perception associated with early AD (El Haj and Kapogiannis 2016). Timing and memory deficits are commonly associated with hippocampal dysfunction. Research emphasizes the role of the hippocampus in distinguishing durations, representing temporal sequences, and encoding the passage of time (MacDonald et al. 2014; Meck et al. 2013). Most existing studies have relied on verbal time‐estimation tasks, with only a few exploring time reproduction or bisection tasks (El Haj and Kapogiannis 2016; Schaffner et al. 2022; Pai et al. 2021).

According to Porter et al., the processing of complex motions, which are dynamic, moving stimuli, is more susceptible to deterioration than that of complex static forms, possibly due to its greater reliance on extensive neural connections, which are considerably affected by AD pathology (Porter et al. 2017). Moreover, distinguishing AD from normal aging might be more effectively achieved through complex motion detection rather than focusing on directionality (Liu et al. 2019; Wu et al. 2020). To better understand the differences in temporal judgment in AD, further research is required to evaluate a broad range of timing tasks, such as the Time–Wall task, which incorporates both time and motion processing.

Performance of the mental rotation task, as measured by accuracy scores and response times, demonstrated notable differences between the CC and those with MCI or MD. No marked differences were observed between the MCI and MD cohorts. The diagnostic accuracy of mental rotation was lower than expected, and the inclusion of the mental rotation task resulted in a multinomial logistic regression model that did not improve cohort classification. According to Suzuki et al., the mental rotation task with a 180° rotating angle was able to distinguish MCI cases from their controls with 80.0% accuracy (Suzuki et al. 2018).

Several factors may explain the limited diagnostic potential of this task. Most notably, constraints on the length and complexity of mental rotation tasks, particularly for older adults and patients with AD, necessitate accounting for fatigue, which can negatively affect performance during extended tasks across all population groups (Ren et al. 2019). In our study, participants completed six practice trials and 32 experimental trials involving mental rotation. The average task duration was 5–10 min for CC and 10–20 min for MCI and MD, with a time limit of 30 min. By contrast, the original mental rotation task within the PEBL battery comprised 128 trials, and the task duration in the study by Langner et al. was approximately 4 h 30 min (Langner et al. 2023). Second, according to Yassa et al., asymptomatic individuals at risk for familial late‐onset AD exhibit more extensive neural activity than healthy individuals during mental rotation tasks. This infers that a genetic predisposition to AD is associated with altered brain function, marked by increased activation in the parietal cortex, particularly in the prefrontal regions, potentially reflecting a compensatory network (Yassa et al. 2008). These results indicate that the diagnostic accuracy of the mental rotation task in distinguishing between cognitively healthy individuals and those with pathology may be influenced by compensatory mechanisms in frontal lobe regions that are not yet affected by AD (Yassa et al. 2008).

While studies have frequently shown that males outperform females on spatial ability tests, our study found no distinct differences in sex when independently analyzing the cohorts (Wei et al. 2016). Furthermore, the mental rotation task performance exhibited a weak correlation with age and educational attainment, suggesting a minimal impact of demographic variables. These findings highlight the need for a more comprehensive evaluation of mental rotation tasks to better capture compensatory brain network function.

This study had several limitations. First, CSF biomarker data were available for only a small number of participants due to the invasive nature of the procedure, and positron emission tomography biomarkers were not used. Including CSF biomarkers for all participants would have enabled the exclusion of MCI caused by dementia with Lewy bodies. This condition can be challenging to differentiate from MCI due to AD when relying on cognitive tests focused on motion perception or mental rotation. Nevertheless, none of the participants met the 2017 revised criteria for the clinical diagnosis of dementia with Lewy bodies (McKeith et al. 2017). Second, the sample size was adequate to demonstrate notable changes; however, additional studies with larger sample sizes would be advantageous for validating these findings. Third, the cross‐sectional design of the study limited the ability to draw definitive conclusions regarding AD progression. Despite the promising results of this study, longitudinal studies are necessary to establish the effectiveness of the Time–Wall and mental rotation tests as reliable assessment tools.

The strengths of this study included the ability to distinguish temporal judgment and motion perception from language skills using the computerized PEBL test. This approach enhanced our understanding of early AD‐related changes and demonstrated strong diagnostic accuracy.

## 5 Conclusions

The Time–Wall task is a freely available and efficient clinical assessment tool, enabling a quick and straightforward evaluation of temporal judgment and motion perception in early AD. We recommend incorporating the Time–Wall task as an additional tool to enhance the early diagnosis of AD. Notably, age did not influence the performance of this task. The mental rotation task exhibited low sensitivity and requires further evaluation, as it may offer insights into compensatory brain network functions. Combining multiple diagnostic tools, including temporal judgment and motion perception assessments, could provide the most accurate and timely AD diagnosis.

## Author Contributions

VS is responsible for the conceptualization of the manuscript; in addition to conducting investigations, formal analyses, and data curation. GP‐K and EA contributed to data curation; in addition to conceptualization of, reviewing, and editing the manuscript. JK contributed to data curation; reviewing, and editing the manuscript. GK provided supervision and contributed to formal analysis, in addition to conceptualization of, reviewing, and editing the manuscript. All authors read and approved the final manuscript.

## Ethics Statement

This study was approved by the Vilnius Regional Bioethics Committee on January 11, 2022 (Approval Number: 2022/1‐1405‐877). Moreover, this study was conducted in accordance with the principles of the Declaration of Helsinki and Standards for Reporting Diagnostic Accuracy Studies (STARD) guidelines. All participants provided written informed consent prior to study participation.

## Consent

All participants provided written informed consent prior to study participation for publication of their de‐identified data.

## Conflicts of Interest

The authors declare that they have no competing interests.

## Funding

The authors have nothing to report.

## Supporting information



Abbreviations: CC = control cohort; MD = mild dementia; MCI = mild cognitive impairment.

Abbreviations: CC = control cohort; MD = mild dementia; MCI = mild cognitive impairment.

## Data Availability

The datasets used and/or analyzed during the current study are available from the corresponding author on reasonable request.
