# Supplementary figures and images for: Time/Movement Estimation and Mental Rotation Tasks as Early Cognitive Markers in Alzheimer's Disease
Source: Brain Behav. 2025 Nov 19;15(11):e71077. doi: 10.1002/brb3.71077 (PMC12631017; doi:10.1002/brb3.71077)

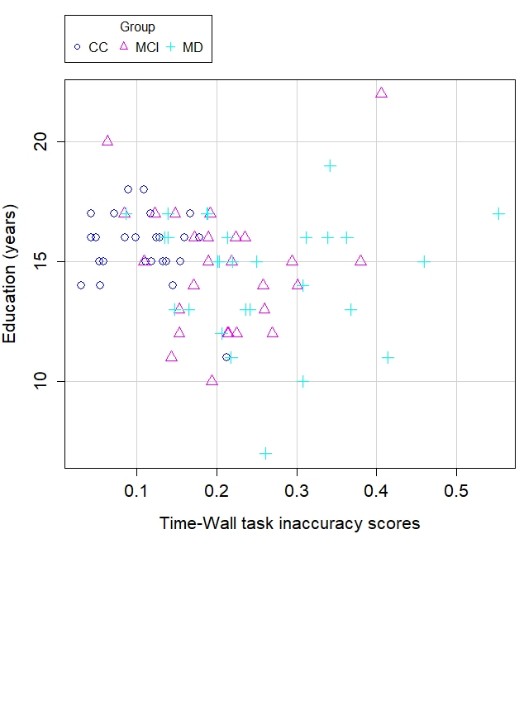

Supplement: Supplementary file 1 — Abbreviations: CC = control cohort; MD = mild dementia; MCI = mild cognitive impairment. [file BRB3-15-e71077-s001.jpg]

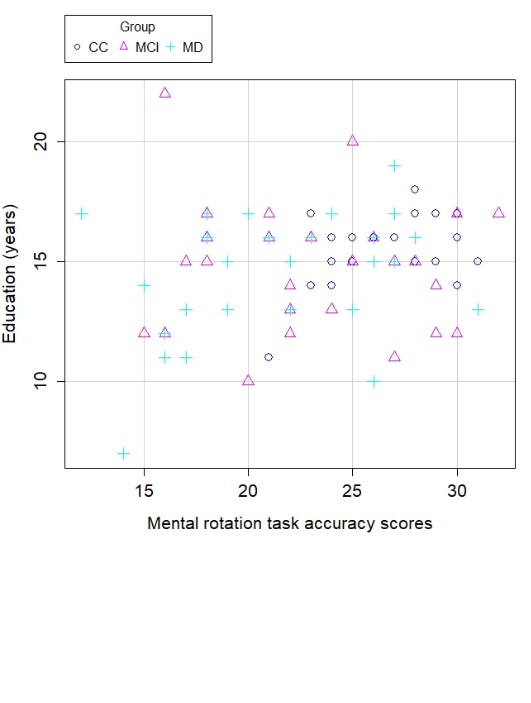

Supplement: Supplementary file 2 — Abbreviations: CC = control cohort; MD = mild dementia; MCI = mild cognitive impairment. [file BRB3-15-e71077-s002.jpg]
